# Mechanism of Intervertebral Disc Degeneration via the β‐Catenin/CCL2 Pathway in Sox9 Conditional Knockout Mice

**DOI:** 10.1002/jsp2.70053

**Published:** 2025-02-26

**Authors:** Khaled Aboushaala, Ana Chee, Frank Ko, Jad Alkhudari, Saurav Sumughan, Howard S. An, Dino Samartzis, Chun‐do Oh

**Affiliations:** ^1^ Department of Orthopedic Surgery Rush University Medical Center Chicago Illinois USA; ^2^ Department of Anatomy and Cell Biology Rush University Medical Center Chicago Illinois USA

**Keywords:** β‐catenin, back pain, CCL2, disc degeneration, mechanisms, molecular, SOX9, spine

## Abstract

**Introduction:**

Degenerative changes in the intervertebral disc (IVD) are known to be a main cause of low back pain (LBP), oftentimes necessitating interventions that may or may not be successful due to a lack of understanding in the degenerative phenotype and its mechanisms. Understanding the molecular mechanisms of disc degeneration can help design new therapies to induce disc regeneration and reduce back pain. This work aimed to understand the effects of conditional deletion of Sox9 in aggrecan‐expressing cells on intervertebral disc degeneration and its underlying mechanisms in mice.

**Methods:**

This study utilized *Agc1‐CreERT2;Sox9* ^*flox/flox*^ mice to investigate the effects of SOX9 deletion on IVD degeneration and associated pain behaviors. Mice were administered tamoxifen to induce conditional gene deletion of Sox9. Structural and degenerative phenotypes of the spine were assessed by a histological scoring system and micro‐computed tomography (microCT). Pain behaviors were evaluated through mechanical allodynia testing and the LABORAS system for spontaneous behavior assessment. Immunohistochemistry identified the expression of proteins of interest, which were further examined by Western blotting. Lastly, quantitative real‐time PCR and promoter assays on IVD cells were used to examine inflammatory and signaling pathways induced by Sox9 deletion.

**Results:**

Crossing *Agc1‐CreERT2* mice with *Sox9* ^*flox/flox*^ mice revealed that *Sox9* conditional deletion (*Sox9* ^*cKO*^) in cartilage tissues causes IVD degeneration and pain behavior. *Sox9* ^*cKO*^ mice spines had narrowed intervertebral disc spaces and disorganized IVD tissues. *Sox9* deletion also increased β‐catenin, C‐C motif chemokine ligand 2 (CCL2), and Glial cell line‐derived neurotrophic factor (GDNF) expression in the IVD, suggesting their roles in disc pain and degeneration and the importance of the β‐catenin/CCL2 pathway in these processes.

**Conclusions:**

Deletion of *Sox9* in Aggrecan‐expressing IVD tissues affects disc degeneration and associated pain behaviors through the β–catenin–CCL2 pathway. Such findings can lead to more targeted, personalized therapeutics in the future to address discogenic origins of LBP.

## Graphical abstract

This study investigates the mechanism of intervertebral disc degeneration via the β‐catenin/CCL2 pathway in Sox9 conditional knockout mice. Our findings reveal that the disruption of Sox9 enhances β‐catenin activity and CCL2 expression, contributing to disc degeneration.

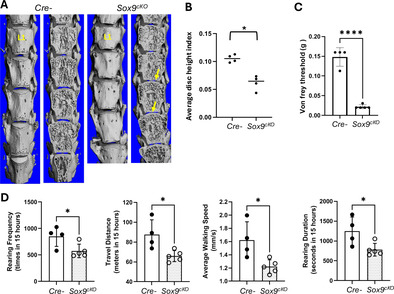

## 1 Introduction

Low back pain is recognized as one of the most prevalent and disabling conditions worldwide, affecting an estimated 80% of the global population with tremendous socioeconomic consequences [1, 2, 3, 4, 5]. Given this prevalence and impact, the development of effective therapies has been a longstanding pursuit, and a thorough understanding of pain mechanisms is essential. Although low back pain is multifactorial, intervertebral disc degeneration is a strong etiological factor. The intervertebral disc is composed of a collagen type II matrix dispersed within a proteoglycan‐rich nucleus pulposus (NP) at the center, and a surrounding collagenous annulus fibrosus (AF) [6, 7, 8]. Between the intervertebral discs and vertebrae are interfaces called the vertebral endplates (EPs), which are composed of a bony layer and a cartilaginous layer [9]. The main proteoglycan of NP is aggrecan, whose protein is a direct target of SOX9, as is collagen type II in the NP [10]. A consequence of intervertebral disc degeneration can lead to “discogenic” origins of pain. This process can be characterized by fissuring or tears of the AF, promoting neurovascular infiltration, disc space narrowing, and perhaps disc bulging or herniation, causing displacement of the disc material and potential compression of the neural elements (e.g., spinal cord, thecal sac, and exiting nerve roots). This process may lead to inflammation in the disc as well as between the disc and the neural interface that may further contribute to the overall symptomology [11, 12].

During the development of the mammalian skeleton, chondrocyte differentiation is regulated by SOX9, one of the SRY‐related HMG‐box‐containing proteins, a transcription factor [13]. SOX9 directly targets several key genes, including *Col2a1* [14, 15], *Col11a2* [16], *Acan* [10], *CD‐rap* [17], *Ctgf* [18], and so forth, which are essential for chondrocyte differentiation [19]. Heterozygous mutations in *Sox9* cause Campomelic Dysplasia (CD), a generalized disease of cartilage characterized by hypoplasia of endochondral bones [20, 21]. This phenotype has also been observed in mouse models of conditional inactivation of the *Sox9* gene [22, 23].

Since the main components of the intervertebral disc are aggrecan and collagens, SOX9 is considered an essential transcription factor for the proper development and maintenance of the intervertebral disc [24, 25, 26]. One of the radiologic manifestations of Campomelic Dysplasia (CD) includes short and somewhat flattened vertebrae [27]. In addition, the postnatal conditional inactivation of *Sox9* leads to intervertebral disc degeneration [28].

Furthermore, as skeletal progenitors differentiate into chondrocytes or osteoblasts, SOX9 acts as a master regulator of chondrocyte differentiation. It is worth noting that in this segregation process, β‐catenin, the functional factor of canonical Wnt‐signaling, is inhibited by SOX9 [29, 30, 31]. A recent detailed study employing postnatal conditional *Sox9* knockout models has demonstrated that *Sox9* deletion leads to disc degeneration and induces or suppresses genes related to the Wnt/LRP6 signaling, inflammation, chemokines, and extracellular matrix proteins [32]. However, whether these molecular mechanisms are associated with pain has not been examined. In this study, we therefore employed the *Sox9* conditional knockout mouse to elucidate the relationship between intervertebral disc degeneration and pain behavior, as well as to identify the specific signaling pathways implicated in this process. Our findings provide novel insights into the pathophysiology of intervertebral disc degeneration and its associated pain. We demonstrate that the *Sox9* regulation of β‐catenin, CCL2, and GDNF constitutes a pivotal mechanism in pain signaling, suggesting that modulation of this pathway may represent a promising therapeutic strategy for intervertebral disc degeneration and its concomitant pain.

## 2 Materials and Methods

### 2.1 Mice and Deletion of SOX9 Using Agc1‐CreERT2; *Sox9* ^*flox/flox*^

*Agc1‐CreERT2* mice with *ROSA* ^*mTmG*^ reporter (#019148, #007676) were obtained from Jackson Laboratory (Bar Harbor, ME, USA). The *Sox9* ^*flox/flox*^ mice (#013106) were described previously [18]. To assess the expression of *Agc1‐CreERT2* in the intervertebral disc, *Agc1‐CreERT2;ROSA* ^*mTmG*^ mice were administered by intraperitoneal (I.P.) injection (1 mg/10 g body weight for 5 consecutive days) with tamoxifen (Sigma–Aldrich Inc.) at 2 weeks of age and sacrificed at the age of 4 weeks. To determine the effects of Sox9 deletion on the intervertebral disc, *Agc1‐CreERT2* mice were crossed with *Sox9* ^*flox/flox*^ mice. The resulting *Agc1‐CreERT2;Sox9* ^*flox/flox*^ mice were administered tamoxifen (1 mg/10 g body weight IP, daily for 5 days) at 8 weeks of age and sacrificed at the age of 16 weeks, presented as *Sox9* ^*cKO*^. In the control group, which consisted of *Cre*‐negative mice, corn oil was injected intraperitoneally using the identical volume. The experimental and control groups were not randomly assigned but instead allocated according to the genotype. All the subsequent procedures and downstream analyses were performed by blinded observers. The animal protocol of this study has been approved by the IACUC of Rush University, and all experimental methods and procedures were carried out in accordance with the approved guidelines. Mice were maintained in a pathogen‐free facility, subjected to a 12/12 h light/dark cycle, and had ad libitum access to standard laboratory rodent chow and water.

### 2.2 Histological Analysis

**FIGURE 1:**
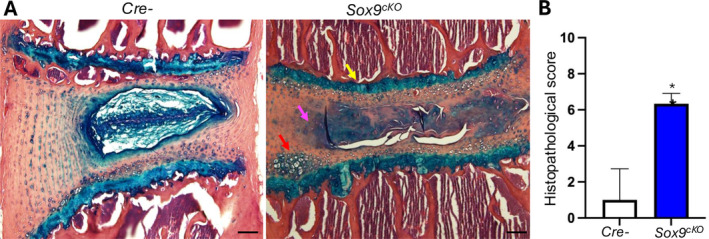
Deletion of Sox9 leads to severe disc defects. (A) Histological analysis (Alcian blue/H&E staining, L4/5, *Cre−*, *n* = 3, *Sox9* ^*cKO*^, *n* = 5, scale bar 100 μm) showing that growth plate cartilage was slightly narrow (yellow arrow). Disc tissue including NP and AF was dramatically disorganized (pink arrow) and hypertrophic cells were shown adjacent to end plate area (red arrow). Agc‐CreER mice were bred with Sox9 flox/fox mice. The resulting *Agc1‐CreER;Sox9 floxflox* mice were administered with tamoxifen (1 mg/10 g body weight IP, daily for 5 days) at 8 weeks of age and sacrificed at the ages of 16‐week‐old, presented as *Sox9* ^*cKO*^. (B) Histological grading assessment was quantitatively measured and represent mean ± SD. Statistical analysis was conducted using two tailed unpaired *t*‐test. *p < 0.05.

The disc tissue was dissected from 4‐month‐old *Cre*‐negative (*Cre−*) and *Agc1‐CreERT2;Sox9* ^*flox/flox*^ (*Sox9* ^*cKO*^) mice from the 2 experimental groups described in Figure 1. Samples were fixed in 10% formalin, decalcified in 14% EDTA, and embedded in paraffin. Paraffin‐embedded samples were sectioned at 8 μm thickness. Histologic/structural changes in disc tissue (L4/5) were evaluated by Alcian blue/hematoxylin and eosin (AB/H&E).

Histological grading was determined using the scoring methods described previously [33]. Tamoxifen induction was performed when mice were at 8 weeks of age. For the histomorphometric analysis, NP, AF, and cartilage endplate areas of 4‐month‐old mice were analyzed.

### 2.3 Plasmid Construction and Reporter Assay

To show a direct effect of the β‐catenin/TCF pathway on *Ccl2* expression, we have generated the human *Ccl2* promoter construct by subcloning a 3.8 kb fragment of the 5′ promoter region (GI access number; 516772) into the firefly luciferase reporter plasmid pGL‐3 (Promega) [34].

This human *Ccl2* promoter contains a consensus β‐catenin/TCF binding site (5′‐CTTTGTA‐3′) located 1,399 bp upstream of the transcription initiation start. A mutated human *Ccl2* promoter reporter construct was generated by mutating the β‐catenin/TCF binding site (5′‐CTTTGGC‐3′). Transient transfection experiments were conducted using Fugene‐6 transfection reagent (Roche Diagnostic) on rat immortalized NP and AF cells. Twenty‐four hours post‐transfection using the wild‐type or the mutated rat *Ccl2* promoter, the cells were treated with 1 μM BIO (the small molecule glycogen synthase kinase‐3 inhibitor, and 6‐bromoindirubin‐3′‐oxime) to activate β‐catenin for 24 h [26, 34]. For each experiment, firefly luciferase activity was normalized to the renilla luciferase activity, which served as an internal control. The results were expressed as fold changes, determined by normalizing each firefly luciferase value to the renilla luciferase internal control value. Each experiment was performed in triplicate and repeated at least three times. Since the results from NP and AF cells are similar, we present the data collectively as disc cells in Figure 5E.

### 2.4 Micro‐Computed Tomography (mCT) Analysis

Formalin‐fixed spine tissues from 4‐month‐old mice from the 2 experimental groups were evaluated by μCT using a Scanco μCT35 scanner. Spine samples were scanned at 12‐μm resolution with a slice increment of 10‐μm. Images from each group were reconstructed at identical thresholds to allow for a 3‐dimensional structural rendering of each spine sample.

Changes in disc space height L3/4 and L4/5 of 4‐month‐old mice were measured. The average Disc height index (DHI) was determined by measurements obtained from the anterior, middle, and posterior portions of the IVD and then dividing this average by the mean height of the adjacent vertebral bodies, as described previously using radiographs [35, 36].

### 2.5 Pain Assessment

The mechanical allodynia test was performed using a calibrated set of von Frey filaments (North Coast Medical Inc., CA, USA). Pain‐related behavior tests were performed in 3‐month‐old mice. Prior to the von Frey hind paw test, the mice were allowed to accommodate for 15 min on a wire mesh grid. The filaments (typical force range used in mice is from 0.04 to 6.0 g, beginning with 0.4 g) were applied to the plantar surface of the hind paw to determine the 50% force withdrawal threshold using the classical up‐down iterative method as previously described [37]. The tests were performed in a blinded manner. The assessment of spontaneous behavior was measured by the Laboratory Animal Behavior Observation Registration and Analysis System (LABORAS, Metris, the Netherlands). Briefly, after the animals were weighed, the mice were placed in separate platforms for 15 h from 18:00 p.m. to 9:00 a.m. the next day. The following parameters were assessed: distance of locomotion, average speed of locomotion, rearing frequency, and rearing duration.

### 2.6 Immunohistochemistry

The disc tissue was dissected from *Cre*‐negative (*Cre−*) and *Agc1‐CreERT2;Sox9* ^*flox/flox*^ (*Sox9* ^*cKO*^) mice. Samples were fixed in 10% formalin, decalcified in 14% EDTA, and embedded in paraffin. Paraffin sections (8 μM) were boiled for 15 min in citrate buffer to reverse cross‐links and unmask epitopes. Sections were blocked with 5% goat serum for 1 h and incubated with anti‐β‐catenin (Abcam, #ab16051) or anti‐CCL2 (Novus, #NB2‐22115) or anti‐GDNF (Santa Cruz, #sc‐13147) at a dilution of 1:200 overnight at 4°C. The following day, sections were incubated with biotinylated goat anti‐rabbit or anti‐mouse antibody for 30 min, followed by incubation with the VECTASTAIN Elite ABC Reagent. ImmPACT DAB Peroxidase Substrate was used to reveal IHC signals, which were then analyzed with the ImageJ program. We stained all disc levels; however, L4/5 was selected as the representative data for the figures and analysis. To address the potential issue of non‐specific binding of the secondary anti‐mouse antibody to endogenous mouse IgG, we compared staining results with and without the primary antibody while using the secondary anti‐mouse antibody. The absence of staining in the no‐primary antibody control confirms that our staining is specific for the primary antibody.

### 2.7 Cell Cultures

The immortalized rat NP and AF cells were cultured with Dulbecco's modified Eagle's medium (DMEM)/Ham's F‐12 (1:1) (Gibco, Gaithersburg, MD) supplemented with 20% fetal bovine serum (FBS) (Gibco, Gaithersburg, MD) and 1% (vol/vol) penicillin and streptomycin in a 37°C, 5% CO_2_ atmosphere [26]. The cell lines were treated with 1 μM BIO or 5 ng/mL IL‐1β for 24 h; then, RT‐qPCR and western blot were performed.

### 2.8 Western Blot Analysis

After washing with PBS twice, immortalized cells were lysed using RIPA buffer (10 mM Tri‐HCl, pH 7.4, 0.01% sodium dodecyl sulfate (SDS), and 0.1% Nonidet P‐40 with protease inhibitors). The lysate protein concentration was measured by BCA protein assay and standardized by total protein using sample buffer (Bio‐Rad, Hercules, CA, USA). The samples were separated by SDS‐10% polyacrylamide gel electrophoresis and transferred to a nitrocellulose membrane. The membrane was blocked with milk in PBS, treated with primary antibody, washed, and incubated with horseradish peroxidase‐conjugated secondary antibody. The primary antibodies, mouse anti‐β‐catenin antibody (Abcam, #ab16051) at a 1:100 dilution and mouse anti‐β‐actin antibody (Santa Cruz, #sc‐517582) at a 1:200 dilution, were incubated with the membranes for 2 h. After washes, the protein bands were visualized by an ECL detection kit (Thermofisher Scientific) under x‐ray exposure.

### 2.9 Quantitative Real Time qPCR

Total RNA was extracted from rat NP or AF cells and analyzed by RT‐qPCR [26] using the RNeasy Plus Mini Kit (QIAGEN) according to the manufacturer's protocol. cDNA was prepared from the mRNA using the iScript cDNA synthesis kit (Bio‐rad). Quantitative polymerase chain reaction (qPCR) was performed using primers specific for each RNA, SYBR Master Mix, and a BIO‐rad CFX96 system. The difference in Ct values (delta Ct) between the Ct value of each sample and that of GAPDH was calculated. The delta Ct value of each gene was then compared to that of each sample. Comparison of mRNA expression of specific genes between control and BIO treatment were performed. Specific PCR primer names and sequences for real‐time PCR are listed in Table 1.

**TABLE 1:** Primer sequences for PCR.

Primers	Forward (5′‐3′)	Reverse (5′‐3′)
Ccl2	GAATGAGTAGCAGCAGGTGAG	ATCTCTCTTCCTCCACCACTA
Ccl3	GGAATTTGCCGTCCATAGGA	GCCCTTGCTGTTCTTCTCT
Ccl5	GGAGAGGTAGGCAAAGCAG	CATCTTCCACAGTCTCTGCTTC
CCR1	GGAACTGGTCAGGAACAATAGC	GAACCTTGAATTCATAAAGACTCTCA
CCR2	GACCGAGTGAGCTCAACATTT	AACCCAACTGAGACTTCTTGC
IL‐1β	CACCTTCTTTTCCTTCATCTTTG	GTCGTTGCTTGTCTCTCCTTGTA
Tnf‐α	ACTGAACTTCGGGGTGATTG	GCTTGGTGGTTTGCTACGAC
Mmp13	GCAGCTCCAAAGGCTACAA	CATCATCTGGGAGCATGAAA
Gapdh	CCACAGTCCATGCCATCAC	TCCACCACCCTGTTGCTGTA

### 2.10 Statistical Analysis

Sample size estimates were based on our prior studies [26] and the statistical analyses were done in consultation with the Rush University Biostatistics Core. We used student's *t*‐test or ANOVA to compare the experimental groups. When *p* < 0.05 from ANOVA, we performed Tukey's HSD post‐hoc comparisons. A similar approach was used for the molecular data. All analyses were performed using the statistical software GraphPad Prism version 10.2.1.(331).

## 3 Results

### 3.1 *Agc1‐CreERT2* Targets Different Types of Tissues in the Intervertebral Disc

To determine whether *Agc1‐CreERT2* works in the presence of tamoxifen, *Agc1*‐*CreERT2* mice were bred with ROSA^*mTmG*^ reporter mice to generate *Agc1*‐*CreERT2*;ROSA^*mTmG*^ mice. ROSA^*mTmG*^ mice expressed red fluorescence in various parts of the intervertebral disc and vertebra, including the growth plate (GP), NP, and AF (Figure S1, Left Panel). After tamoxifen administration to 2‐week‐old *Agc1*‐*CreERT2*;ROSA^*mTmG*^ mice, enhanced green fluorescent protein (*mG*) exhibiting green fluorescence was observed in the growth plate (GP), NP, and inner AF at 4‐week‐old mice, indicating the *Agc1‐CreER* activation and the successful deletion of the floxed stop codon of tdTomato (*mT*) genes in regions where Aggrecan is expressed (Figure S1, Right Panel). In addition, the EP tissue also showed green fluorescence but was less intense compared to other disc tissues. This supports our use of Agc1‐CreERT2 to conditionally delete Sox9 in the intervertebral disc to explore the signaling pathways involved in disc degeneration and associated pain mechanisms.

### 3.2 Deletion of Sox9 Leads to Severe Defects in the Intervertebral Disc

*Agc1‐CreERT2/Sox9* ^*flox/flox*^ mice were treated with tamoxifen at 8 weeks of age and sacrificed at 16 weeks of age to generate *Sox9* conditional knockout (*Sox9* ^*cKO*^) in tissues where aggrecan is expressed. The harvested tissues, including the intervertebral disc, were stained with Alcian blue/H&E. The growth plate cartilage in the *Sox9* conditional knockout mice was slightly narrower compared to the control (Figure 1A). Specifically, the NP tissue in *Agc1‐CreERT2/Sox9* ^*flox/flox*^ mice exhibited a fibrous structure, reduced cell numbers, empty lacunae without cells, and severe matrix disorganization. Furthermore, the disc tissue, encompassing the NP and AF, exhibited significant disorganization and the presence of hypertrophic cells near the EP area. The AF tissue displayed a marked loss of normal lamellar organization, and the interface region showed undefined boundaries between the NP–AF and EP–AF, suggesting that *Sox9* conditional deletion leads to intervertebral disc degeneration.

Histopathological grading was assessed and quantitated in Figure 1B, and microfractures in the NP and AF tissue were not scored due to artifacts introduced during histological preparation.

### 3.3 The Narrowing of the Intervertebral Disc and Pain Behavior Were Observed in Sox9 Conditional Knockout Mice

**FIGURE 2:**
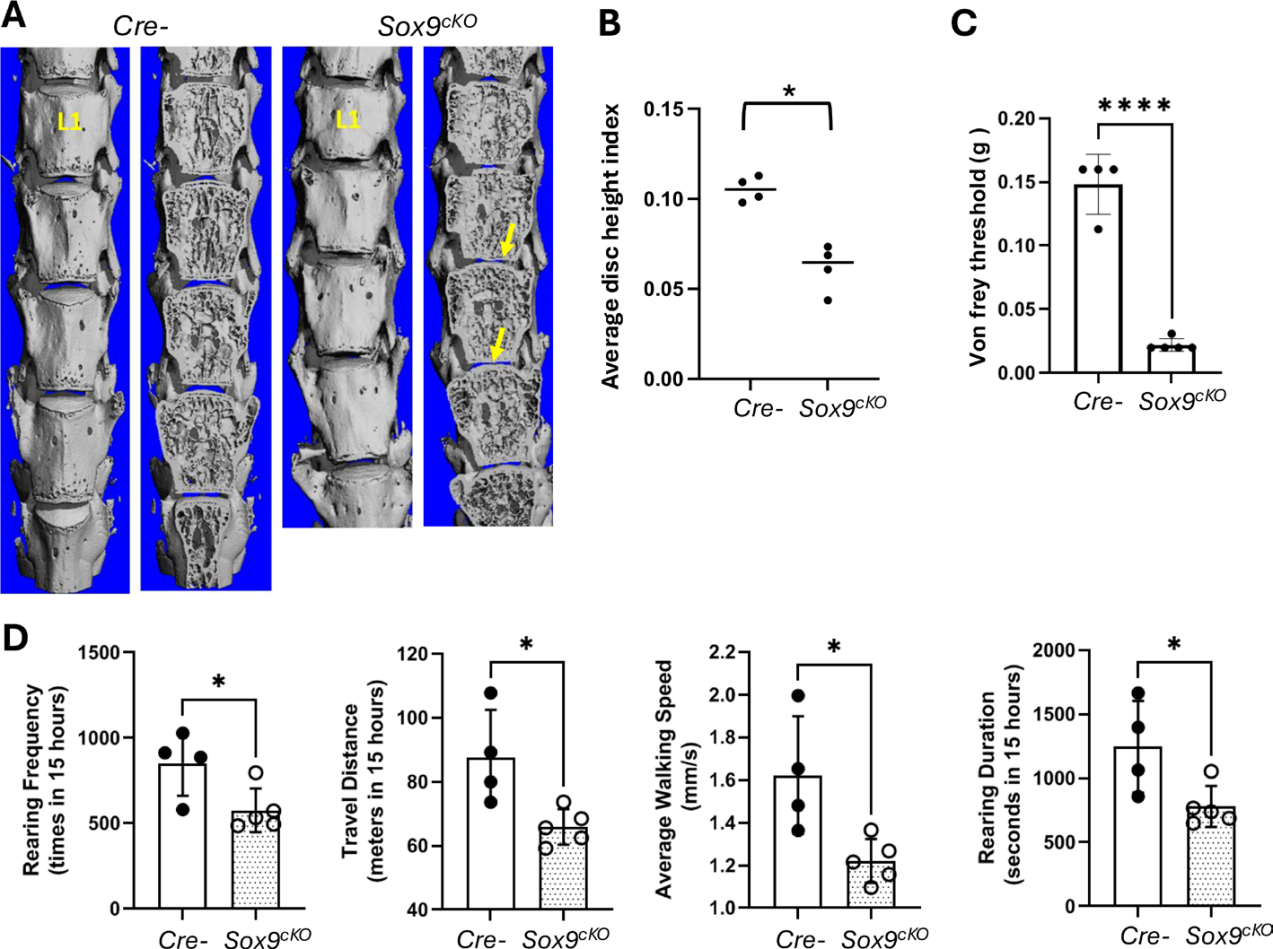
Disc defect and pain behavior were observed in Sox9 deletion mice. (A, B) μCT analyses were performed in 2 groups of mice; (1) *Cre−*, (2) *Sox9* ^*cKO*^ mice. Deletion of Sox9 showed the phenotype of disc space narrowing (yellow arrows). (B) Average disc height index was calculated by measuring the disc height of L3/4 and L4/5 (*Cre−*, *n* = 4, *Sox9* ^*cKO*^, *n* = 4). (C, D) Pain‐related behavior tests were performed in 3‐month‐old mice. The results of Von frey threshold, rearing frequency, travel distance, average walking speed and rearing duration showed that *Sox‐9* deletion mice have high pain sensitivity (*Cre−*, *n* = 4, *Sox9* ^*cKO*^, *n* = 5). *p < 0.05, ****p < 0.01, two‐way ANOVA followed by Turkey HSD test, compared to *Cre*‐negative control mice.

Micro‐CT representative images qualitatively showed that the disc space was reduced in *Sox9* ^*cKO*^ mice (Figure 2A). We quantitatively measured the average disc height and found significant reductions in DHI indices (Figure 2B). As shown in Figure 2C, *Sox9* ^*cKO*^ mice (*n* = 5) demonstrated significantly lower withdrawal thresholds compared to control mice (*n* = 5), indicating increased sensitivity to mechanical stimuli (*p < 0.01). Moreover, the LABORAS assessment revealed significant differences between control mice and *Sox9* ^*cKO*^ mice, specifically reduced rearing frequency, travel distance, average walking speed, and rearing duration (Figure 2D). Subsequently, the molecular mechanisms underlying pain associated with intervertebral disc degeneration due to *Sox9* deletion were examined.

### 3.4 GDNF Expression Increased in the NP and EP Areas in *Sox9* Deletion Mice

**FIGURE 3:**
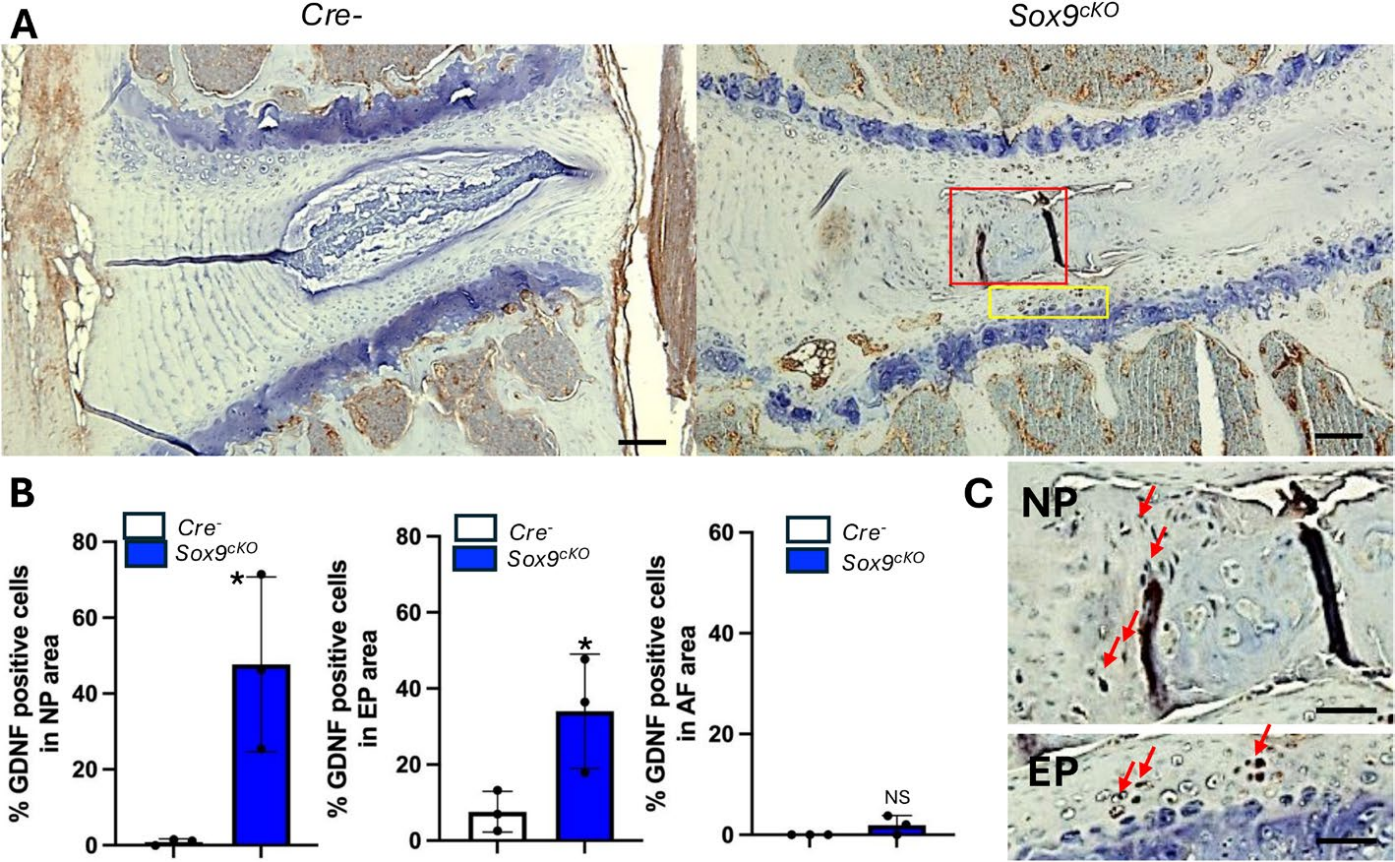
GDNF expression increased in the NP and EP areas in *Sox9* deletion mice. Representative images of immunohistological staining (L4/5, *n* = 3, scale bar 100 μm in A, 25 μm in C) with GDNF showing *Aggrecan* targeting intervertebral disc in two group mice (1) *Cre−*, (2) *Sox9* ^*cKO*^ mice. GDNF‐positive cells were detected in NP and EP areas in *Sox9* deletion mice (A) and magnified images were presented (C). (B) Quantitative measurements represent mean ± SD. Statistical analysis was conducted using a two‐tailed unpaired *t*‐test. *p < 0.05. Arrowheads indicate a positive signal. AF: annulus fibrosus, EP: endplate, NP: nucleus pulposus.

GDNF (Glial‐cell‐line‐derived neurotrophic factor) and its receptors were previously demonstrated to be upregulated in the cell line of human intervertebral discs degenerated by IL‐1β treatment [38]. To investigate the regulation of GDNF during intervertebral disc degeneration and its potential contribution to associated pain, we analyzed its expression in disc tissues. Figure 3A illustrates that GDNF was highly expressed in the NP and EP areas of *Sox9*‐deficient disc tissues. The levels of GDNF expression were quantified across different areas, including NP, EP, and AF, and the results are depicted in Figure 3B. Detailed visualizations of GDNF expression in these specific regions are provided in the high‐magnification images of Figure 3C.

### 3.5 Activation of β‐Catenin/CCL2 Is Associated With Intervertebral Disc Degeneration and Increased Pain Behavior

**FIGURE 4:**
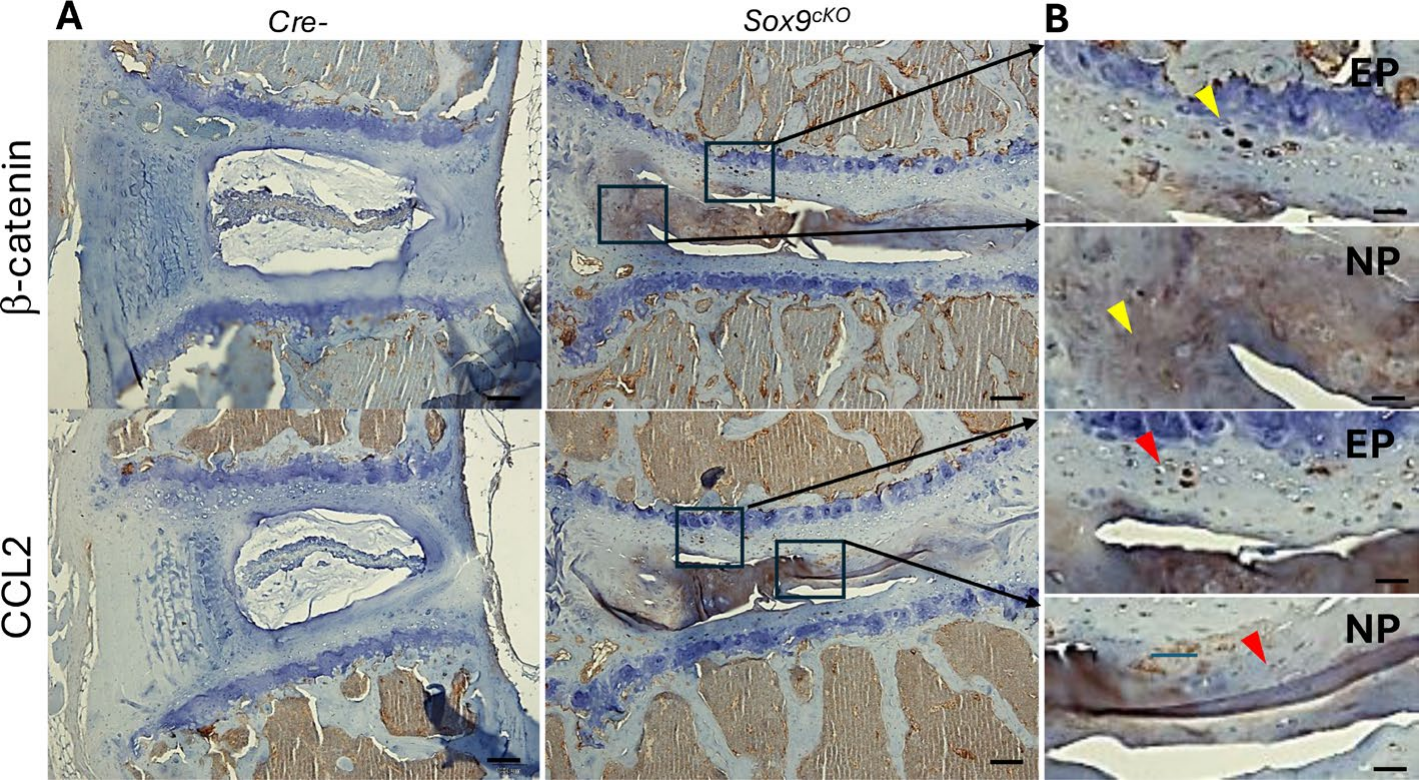
β‐catenin and CCL2 expression was increased in *Sox9* deleted disc tissues. Immunohistochemistry data (L4/5, *n* = 3, scale bar 100 μm in A, 25 μm in B) demonstrated that β‐catenin and CCL2 protein expression was markedly increased (yellow arrows and red arrows, right panel) in disc tissues from 4‐month‐old *Sox9* ^*cKO*^ mice when SOX9 protein expression was not detected by administration with tamoxifen (1 mg/10 g body weight, IP, daily for 5 days) at 8 weeks of age.

**FIGURE 5:**
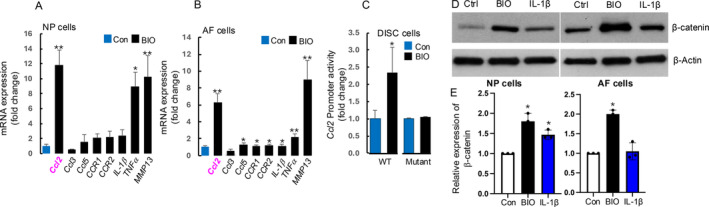
β‐catenin and inflammatory cytokines in rat NP and AF disc cells. (A and B) Treatment of NP and AF cells with BIO (1 μM) significantly up‐regulated *Ccl2, TNF*α, and *Mmp13* gene expression. *p < 0.05, **p < 0.01, unpaired Student *t*‐test, *n* = 3. C. The construct of *Ccl2* promoter reporter harbors a consensus β‐catenin/TCF binding site, 5′➔CTTTGAT‐3′ site located 1082 bp upstream of the transcription initiation start in rat *Ccl2 promoter*. BIO treatment increased *Ccl2* promoter activity in rat disc cells. Mutation of the β‐catenin/TCF binding site (5′‐CTTTGAT‐3′➔ 5′‐CTTTGCG‐3′) abolished BIO‐induced *Ccl2* promoter activity in rat disc cells. *p < 0.05, one‐way ANOVA followed by Tukey HSD test, *n* = 4. (D and E) A GSK‐3β inhibitor, BIO (6‐bromoindirubin‐30‐oxime), and IL‐1β up‐regulated β‐catenin protein levels in NP and AF cells. Rat NP and AF cell lines were treated with BIO (GSK‐3β inhibitor, 1 μM) and IL‐1β (5 ng/mL) for 24 h. Both BIO and IL‐1β significantly up‐regulated β‐catenin protein levels in NP and AF cells.

Since *Sox*9 normally represses the function of β‐catenin in the Wnt signaling pathway during the differentiation of common progenitors into chondrocytes and osteoblasts [23, 31], its deletion may lead to elevated β‐catenin levels in the intervertebral discs. We therefore examined β‐catenin levels in *Sox9*‐deleted intervertebral disc tissue (Figure 4, Upper panel). As expected, a significant increase in β‐catenin was observed in the degenerated intervertebral discs. To determine the molecular mechanism by which β‐catenin regulates downstream target genes, we examined CCL2 expression in Sox9‐deleted intervertebral disc tissue since CCL2 serves as a ligand for the chemokine receptor CCR2, which plays a critical role in leukocyte migration during inflammation [39, 40] and has been shown to play important roles in osteoarthritis (OA) development and OA pain [41]. We found that CCL2 levels were elevated in the degenerated discs (Figures 4 and 5). In addition, previous RNA‐seq analysis comparing *Sox9* deletion with control cells showed a significant upregulation of *Ccl2* expression in Sox9‐deleted chondrocytes (Figure S2) [42]. To elucidate the downstream target genes in disc cells, we then analyzed changes in the expression of multiple genes that have been reported to be involved in disc degeneration [38]. We found that the most significantly upregulated genes by BIO were *Mmp13* and *Ccl2* in AF and NP cells (Figure 5A,B). We then examined if the Wnt/β‐catenin/Tcf4 complex directly upregulates C‐C motif chemokine ligand 2 (*Ccl2*). We cloned a 2.5 kb long rat *Ccl2* promoter and found that there are conserved β‐catenin/TCF binding sites. Treatment of BIO significantly upregulated the *Ccl2* promoter activity, while the upregulation was abolished when the β‐catenin/TCF binding site was mutated (Figure 5C) in disc cells. We treated the cells with BIO (6‐bromoindirubin‐3′oxime), a GSK‐3β inhibitor, and found that BIO, as well as IL‐1β significantly upregulated β‐catenin protein levels in rat AF and NP cells. Additionally, IL‐1β increased the protein and mRNA expression of CCL2 [43], suggesting a potential IL‐1β → β‐catenin → CCL2 signaling pathway in disc cells (Figure 5D,E).

## 4 Discussion

The aim of our study was to investigate the role of Sox9 in maintaining intervertebral disc (IVD) health and to explore how its deletion impacts disc degeneration and pain behaviors. While we acknowledge that Sox9 mutations are not a common cause of IVD degeneration in clinical settings, our Sox9cKO mouse model provides a valuable framework to study the molecular and cellular mechanisms of degeneration, including disrupted matrix organization and cellular dysfunction, which are relevant to natural degenerative processes. MicroCT analysis further indicated intervertebral disc degeneration. All phenotypes were consistent with our previous report [18] and those of other groups employing Sox9 conditional deletion mice [28, 32]. To explore the pain mechanisms related to intervertebral disc degeneration, we performed pain assessments using this mouse model. As shown in Figure 3, *Sox9* ^*cKO*^ mice exhibited a reduction in the von Frey threshold, indicating increased sensitivity to mechanical stimuli. In addition, the LABORAS assessment revealed significant differences between *Cre−* and *Sox9* ^*cKO*^ mice, specifically reduced rearing frequency, travel distance, average walking speed, and rearing duration. These findings indicate decreased exploratory behavior, limited overall movement, and impaired locomotor activity. While *Sox9* ^*cKO*^ mice also have knee joint OA and temporomandibular joint OA (data not shown), which could affect these assessments, our primary aim was to identify pain‐related molecular mechanisms. The upregulation of GDNF in degenerated disc tissues suggests its involvement in the pain mechanism associated with disc degeneration in this mouse model (Figure 3). Although many studies have used this mouse model [5, 18, 32], our pain assessment is the first to be reported in *Sox9* ^*cKO*^ mice. To explore the pain mechanisms in intervertebral disc degeneration in this mouse model, a key focus is on the downstream effects of Sox9. Previous evidence suggests that Sox9 plays a pivotal role in regulating β‐catenin expression and activity. Sox9 repression of β‐catenin is necessary for the proper differentiation of mesenchymal cells into chondrocytes [30]. Sox9 can inhibit β‐catenin/TCF transcriptional activity [44] and interacts with the β‐catenin destruction complex, promoting β‐catenin degradation [31]. However, recent reports indicate that Sox9 represses Wnt/β‐catenin signaling independently of the β‐catenin destruction complex by regulating MAML2 [45]. As expected, our study showed that the deletion of Sox9 results in elevated β‐catenin levels in intervertebral discs (Figure 4). Following the examination of β‐catenin expression, we investigated CCL2 due to its intricate link to pain mechanisms.

Previous studies demonstrated that β‐catenin regulates the expression of CCL2 [34, 37], highlighting its importance in inflammatory and pain responses. CCL2, also known as monocyte chemoattractant protein 1 (MCP‐1), plays a crucial role in recruiting monocytes and other immune cells to sites of inflammation, and its elevated levels have been associated with heightened pain sensitivity and neuropathic pain [46, 47, 48]. Recent research has provided evidence that β‐catenin signaling can regulate CCL2 expression in breast cancer cells [34, 49] and in the human chondrocyte cell line [37]. Our results showed that CCL2 is also increased in Sox9‐deleted tissues, suggesting that the dysregulation of the β–catenin–CCL2 pathway due to Sox9 deletion may drive the inflammatory response and pain observed in degenerative disc disease. Next, we confirmed that β‐catenin regulates CCL2 expression in our established disc cell line. Using a reporter construct of the CCL2 promoter, we demonstrated that β‐catenin can regulate CCL2 expression. We anticipate β‐catenin to regulate CCL2 expression by binding to the CCL2 promoter region, as reported in other cell lines [37]. As shown in Figure S1, RNA‐Seq analysis data showed that CCL2 expression is significantly increased in Sox9‐deleted chondrocytes, indicating Sox9's involvement in regulating CCL2 expression.

However, the β‐catenin mRNA level was not dramatically increased according to the RNA‐Seq data. This suggests that Sox9 may regulate β‐catenin at a protein level. The increased CCL2 expression observed in Sox9‐deleted chondrocytes could be influenced by elevated β‐catenin protein levels, despite unchanged mRNA levels. Further, we identify glial cell line–derived neurotrophic factor (GDNF) as a potential mediator of nociceptive hypersensitivity in the context of inflammation (Figure 3). Our data show GDNF upregulation in degenerated discs due to SOX9 deletion. In future studies, to comprehensively analyze pain mechanisms in disc tissues, several additional analyses should be conducted to quantify other key pain‐related molecules such as IL‐1β, TNF‐α, NGF, and BDNF within the disc tissues.

Our study has several major strengths that enhance the understanding of intervertebral disc degeneration and its associated pain mechanisms, particularly the β‐catenin and CCL2 pathways, by utilizing the *Agc1‐CreERT2/Sox9* ^*flox/flox*^ mouse model. However, we also acknowledge the limitations that should be addressed in future studies. First, in our study, we treated mice with tamoxifen at 8 weeks of age. To better model adult mice and potentially observe more pronounced phenotypes, future studies should consider injecting tamoxifen at 12 weeks of age. Despite this difference, our findings on intervertebral disc degeneration are consistent with previous studies [28, 32] that injected tamoxifen at 12 weeks. Second, our mouse model also exhibits knee OA and TMJ OA phenotypes, which could confound the interpretation of pain specifically associated with intervertebral disc degeneration. To address this, future research should focus on analyzing pain signaling in a lumbar spine instability mouse model to directly associate disc degeneration with pain. Finally, to confirm that ectopic expression of Sox9 can prevent inflammation and alleviate pain through the mediation of the β‐catenin/CCL2 pathways, additional experiments are necessary. These should include overexpression studies and functional assays to validate the therapeutic potential of targeting Sox9 in mitigating disc degeneration‐associated pain.

## 5 Conclusions

This study demonstrates that Sox9 deletion leads to severe intervertebral disc degeneration, characterized by disrupted matrix organization and increased inflammation, without mechanical herniation. Elevated β‐catenin and CCL2 levels in Sox9‐deleted tissues suggest that the β‐catenin‐CCL2 pathway plays a crucial role in the inflammatory response and pain associated with disc degeneration. Pain assessments revealed increased sensitivity to mechanical stimuli and decreased locomotor activity in the mouse model. Future studies should focus on adult mice, utilize lumbar spine instability models, and investigate the therapeutic potential of targeting Sox9 to mitigate pain through the β‐catenin/CCL2 pathways.

## Author Contributions

K.A., A.C., H.S.A., and C.‐d.O. contributed to the conception and design of the work. K.A., A.C., F.K., J.A., S.S., and C.‐d.O. contributed to data acquisition and analysis. K.A., A.C., F.K., H.S.A., D.S., and C.‐d.O. contributed to the interpretation of the data and manuscript preparation. All co‐authors have reviewed and approved the final version of this manuscript for publication.

## Conflicts of Interest

The authors declare no conflicts of interest.

## Supporting information


**Figure S1:** *Agc1‐CreERT2* could efficiently target different types of cells in disc tissue. Agc1‐CreERT2 could efficiently target different types of cells in disc tissue (L4/5, *n* = 5, scale bar 100 μm). *Agc1*‐CreERT2 mice were bred with ROSA^*mTmG*^ reporter mice. The resulting *Agc1*‐*CreERT2*;ROSA^*mTmG*^ mice were administered with tamoxifen (1 mg/10 g body weight, IP, daily for 5 days) at 2 weeks of age and sacrificed at the age of 4‐week‐old. The result of fluorescence microscope analysis showed that *Agc1*‐*CreERT2* mice mainly target growth plate cartilage cells (yellow arrowhead), NP cells (white arrowhead), and AF cells (pink arrowhead).
**Figure S2:** *Ccl2* gene expression is significantly increased in *Sox9*‐deleted chondrocytes. RNA‐Seq analysis compared the RNA expression profiles of primary *Sox9* flox/flox mouse chondrocytes infected with Ad‐CMV‐Cre to those infected with a control adenovirus. The results indicated that when the levels of Sox9 mRNA were decreased by infection with Ad‐CMV‐Cre, *Ccl2* expression was significantly increased, as shown by log2 differential expression (DE). However, the level of β‐catenin expression did not significantly change.
